# Study of otoacoustic emissions during the female hormonal cycle

**DOI:** 10.1016/S1808-8694(15)30759-X

**Published:** 2015-10-19

**Authors:** Priscila Oliveira Arruda, Isabella Monteiro de Castro Silva

**Affiliations:** 1 4th year Speech Therapy undergraduate; 2 Master's degree, Adjunct Professor - Planalto University Center, Federal District (Centro Universitario Planalto do Distrito Federal - UNIPLAN)

**Keywords:** hormonal cycle, ciliated cells, otoacoustic emissions

## Abstract

The hormonal changes that occur in a short time span promote modifications all over the woman's body, with physical and emotional manifestations which are frequently observed.

**Aim:**

to evaluate the activity of the external ciliated cells in women during their menstrual cycle, observing the effect of hormonal changes caused by the cycle in their 3 phases.

**Methods:**

this is a longitudinal prospective study where 21 women between 20 and 35 years old who did not take any contraceptive medicine were assessed. Transient otoacoustic emissions were evaluated by distortion product during the 3 phases of the menstrual cycle (luteal, follicular and ovulatory phases). The SPSS 13.0 software was used to analyze the data.

**Results:**

the phases of menstrual cycle do not alter the amplitude and reproducibility values of the transient otoacoustic emissions. We noticed a difference between the ears in the frequency of 1.5 KHz in the amplitude of emissions by distortion product, and the right ear showed the highest values.

**Conclusion:**

There are no significant differences in transient otoacoustic emissions and distortion products in the phases of the menstrual cycle.

## INTRODUCTION

The menstrual cycle is divided into three phases. The follicular phase starts with menstrual bleeding and lasts, on average, 15 days. The ovulatory phase lasts about three days, culminating in ovulation, when an ovule is released. The luteal phase lasts about 13 days and ends with the beginning of menstruation, which begins a new cycle.

In the ovulatory phase, soon after ovulation, progesterone levels increase, and LH/FSH (luteinizing hormone/follicle-stimulating hormone) and estrogen levels decrease. Progesterone levels peak in the luteal phase as LH/FSH levels decrease still further. High progesterone levels may increase sodium, chloride and water reabsorption. The cycle ends when progesterone and LH/FSH levels are at a minimum, after which a new cycle begins.^1^

Hormone level variations in a short timeframe alter the whole female organism, leading to physical and emotional manifestations that may be quite evident. A previous study^2^ found a decrease in the fundamental frequency of voice in women during the premenstrual period. This period is the end of the luteal phase, in which progesterone and LH/FSH levels drop significantly. Homeostasis and the biochemical status of inner ear fluid is essential for balance and for hearing.^3^ Changes in sodium and water reabsorption that take place during the menstrual cycle may affect the function of this part of the peripheral auditory system, and may affect homeostatis, which causes auditory and labyrinthic symptoms.^3^

Various studies have tested the hypothesis that estrogen concentration variations in women change the functional cerebral asymmetry.^4^, ^5^, ^6^ Right-ear dominance in verbal auditory tasks indirectly reveals a left cerebral advantage for dealing with these stimuli. When testing is done across the menstrual cycle, the right ear advantage is observed to decrease in the premenstrual period compared to the postmenstrual period.^6^

The cochlea, located in the inner ear, has an active mechanism that might be subdivided into three separate steps, as follows:

STEP 1: mechanoelectrical transduction of outer hair cells - the stirrup exerts pressure on the oval window, which propagates the resulting vibrations into the perilymph, causing the basilar membrane and the organ of Corti to oscillate. These oscillations deflect outer hair cell stereocilia, which are coupled to the tectorial membrane. Stimulation is frequency-dependent, as the basilar membrane vibrates differently at high frequencies (the oscillation peak is close to the base of the cochlea) and at low frequencies (the oscillation peak is close to the apical turn of the cochlea). Deflections of the stereocilia open potassium channels on the cell membrane, generating receptor electrical potentials or cochlear microphonic potentials.^7^

STEP 2: electromechanical transduction of outer hair cells - the potentials that are generated produce rapid contractions of the outer hair cells in phase with the frequency of a sound stimulus. The rapid contraction mechanism is the basis of the cochlear active amplification process; the cells connect basilar and tectorial membrane vibrations (both vibrating in phase), resulting in amplification of the original source frequency.^7^

STEP 3: mechanoelectrical transduction of inner hair cells - the actively outer hair cell amplified vibration from the basilar and tectorial membranes deflects the longer cilia (in contact with the tectorial membrane) of those inner hair cells located in the region corresponding to the stimulus frequency. This deflection causes potassium to enter the cell, generating a receptor potential that releases neurotransmitters, forming an electrical stimulus corresponding to the sound message.^7^ Information is then transmitted along the acoustic nerve to the central nervous system.

Analysis of otoacoustic emissions (OAE) is a test that reads, in the auditory canal, sounds generated by outer hairy cell contractions following the presentation of a sound stimulus. OAE may be spontaneous or evoked. Spontaneous OAE are found in about 40% of normal-hearing subjects;^8^ evoked OAE are a release of energy picked up in the acoustic canal as a response to acoustic stimuli, which may be a click (transients) or two pure sounds (distortion products).^9^ This test provides information about outer hair cell integrity during the active cochlear mechanism.^10^ A hypothesis suggests that hormonal changes might be responsible for inner ear diseases, such as Ménière's disease.^8^ There is a consensus in the literature that OAE reception is highly stable, which justifies its medical use.^11^^,^^12^ The amplitude of OAE evoked by transient stimuli or distortion products in audiologically normal subjects may vary according to the frequency being tested, or between subjects. Amplitude within a given subject, however, is remarkably consistent.^12^ Changes due to noise, drugs, disease or efferent excitation taking place in minutes may be monitored.^11^

Menstrual cycle variations in sodium and water absorption and reabsorption affect the active mechanism of the cochlea; the amplitude of OAE may be used to assess such variations in each phase of the cycle.^3^ Previous studies of fluctuations in spontaneous OAE frequencies appear to follow the same pattern of estradiol concentration fluctuations during the cycle.^13^ In the normal menstrual cycle the frequency decreases before menstruation and increases immediately after a new cycle begins. Natural or medication-induced anovulation reduces the characteristic OAE fluctuations during the cycle.

The aim of this paper was to assess hair cell activity in women during the menstrual cycle, noting the effects of hormone variations in each of its three phases.

## MATERIAL AND METHOD

The Research Ethics Committee of the Health Sciences School, Brasilia University, approved this study (protocol number 082/2005).

### Participants

Twenty-one women aged between 20 and 35 years (to exclude the effects of aging) not using contraceptive medication or hormones (to avoid data contamination) were included (information was obtained in clinical history-taking).

Participants were connected to the institution as teachers, staff or students, and were evaluated in three consultations with tests in the School Clinic.

### Procedures

Participants were informed about the study and, upon agreement, signed a free informed consent form.

The 21 women were interviewed to collect the history, otological history, information about the use of medication and about their menstrual cycles. Basic audiological assessments were made (meatoscopy and pure tone audiometry).

Upon finding no auditory conditions, an assessment of TOAE and DPOAE was made. These procedures were conducted three times, as follows: during the follicular phase, during the ovulatory phase and during the luteal phase. The aim was to assess cochlear activity in all of the phases of the cycle.

### Material

§ Audiological history of the adult (School Clinic) - adapted
§ Maico audiometer - models MA-41 and MA-42
§ ILO-92 Cochlear Analyzer coupled to a PC
§ Non-acoustically treated room (along a little used aisle)

### Analysis of Responses

TOAE were considered as present when 1 and 1.5 kHz frequency amplitudes were equal to or higher than 3dB and when 2, 3 and 4 kHz frequency amplitudes were equal to or higher than 6dB; in both cases wave reproducibility had to be equal to or higher than 50%. Responses from the 2f1-f2 distortion product OAE sites at 70 dB L1 and L2 signal intensity values, the most robust and well perceived, were analyzed.^10^ DPOAE were considered as present when the difference between amplitude and noise was over 6dB.

The SPSS 13.0 software was used for multivariate data analysis, to observe the effect of independent variables (ear assessed and phase of the menstrual cycle) on OAE (variable dependent).

## RESULTS

Clinical histories and basic audiological testing (meatoscopy and pure tone audiometry) were normal in our entire sample; there were no impediments for OAE testing.

**Chart 1:**
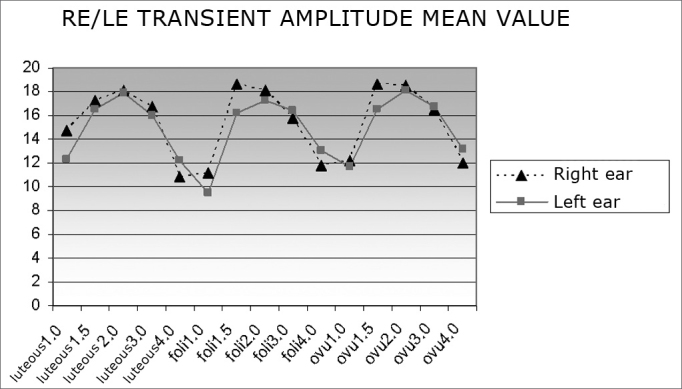
Mean transient OAE amplitudes in both ears according to the phase of the menstrual cycle (luteal, follicular and ovulatory phases).

Chart 1 shows mean transient OAE amplitude values in right and left ears across the menstrual cycle. A decrease in 1 and 4 Hz frequency values may be seen in all three menstrual phases.

**Chart 2:**
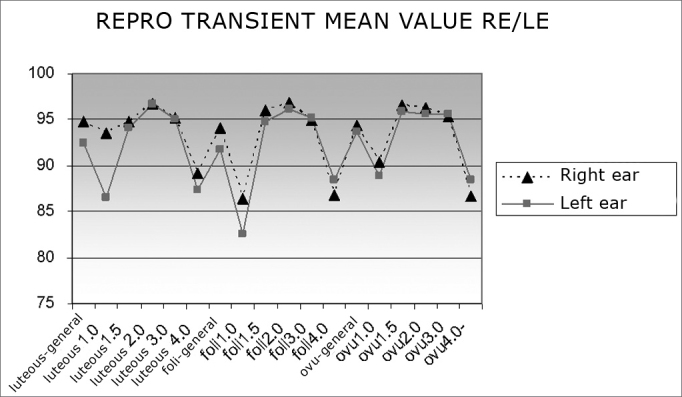
Mean transient OAE reproducibility values in right and left ears according to the phase of the menstrual cycle (luteal, follicular and ovulatory phases).

Chart 2 shows mean transient OAE reproducibility values in right and left ears across the menstrual cycle. A decrease in 1 and 4 Hz frequency values may be seen in all three menstrual phases.

**Chart 3:**
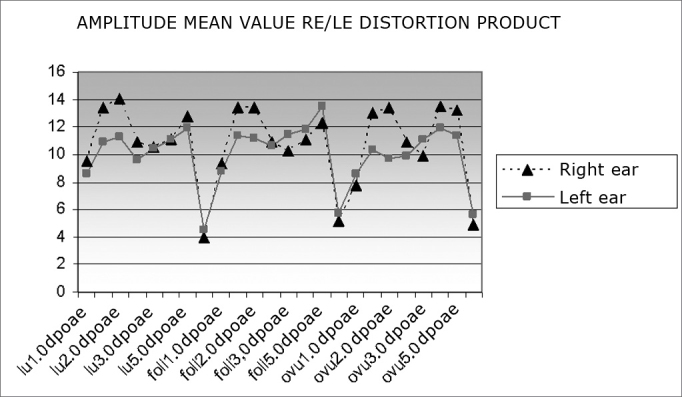
Mean distortion product OAE amplitude values in right and left ears according to the phase of the menstrual cycle (luteal, follicular and ovulatory phases).

Chart 3 shows mean distortion product OAE amplitude values in right and left ears across the menstrual cycle. A sharp decrease in 1 and 6 Hz frequency values may be seen in all three menstrual phases.

**Chart 4:**
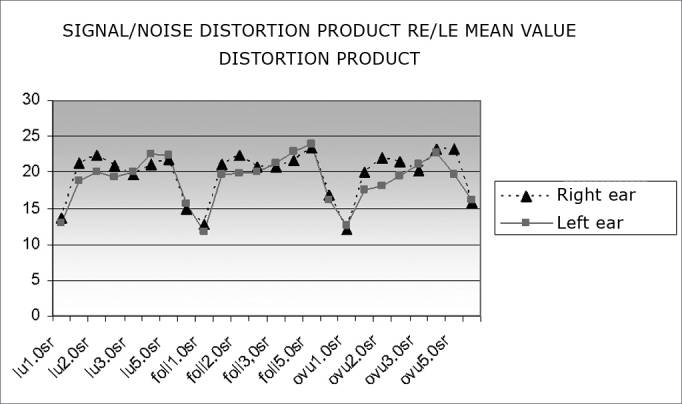
Mean product distortion OEA signal/noise ratio values in right and left ears according to the phase of the menstrual cycle (luteal, follicular and ovulatory phases).

Chart 4 shows the mean product distortion OEA signal/noise ratio values in right and left ears across the menstrual cycle. A sharp decrease in 1 and 6 Hz frequency values may be seen in all three menstrual phases.

Table 1 presents multivariate analysis results showing the effect of each ear on distortion product OAE amplitudes; a single significance value at 1.5 kHz is given, the right ear having the highest values.

**Table 1:** Results of the multivariate analysis - effect of each ear on distortion product OAE amplitudes.

Independent variables	Dependent variables	p
	1000 Hz	0,717
	1500 Hz	0,049*
	2000 Hz	0,055
EAR	2500 Hz	0,526
	3000 Hz	0,473
	4000 Hz	0,948
	5000 Hz	0,741
	6000 Hz	0,686

* Significance - p < 0.05.

Table 2, Table 3 present TOEA and DPOEA means and standard deviations for each ear that was tested according to each menstrual cycle.

**Table 2:** Distribution of TOEA and DPOEA mean values and standard deviations according to each ear that was tested.

TOAE								DPOAE						
		1	1,5	2	3	4	1	1,5	2	2,5	3	4	5	6
dB Mean	OD	12,8	12,3	18,3	16,4	11,7	9	13,5	13,7	10,9	10,1	11,7	12,7	4,51
	OE	11,1	16,5	17,8	16,4	12,8	8,6	10,9	10,7	10,1	11	11,6	12,3	5,29
Standard deviation dB	OD	6,61	6,52	5,76	6,25	5,52	6,6	7,22	7,58	7,1	7,29	7,34	6,67	10,1
	OE	6,78	6,5	6,63	7,02	6,13	5,6	7	8,79	7,46	5,89	6,29	9,44	10,4

**Table 3:** Distribution of TOEA and DPOEA means and standard deviations according to each phase of the menstrual cycle.

TOAE								EOAPD						
		1	1,5	2	3	4	1	1,5	2	2,5	3	4	5	6
Mean value dB	Follicular ovulatory luteous	10,3	17,5	17,7	16,1	12,4	9,3	12,7	12,4	10,8	10,7	11,5	12,8	11,5
		13,5	16,9	18	16,4	11,5	9,1	12,2	12,6	10,3	10,5	10,9	12,4	4,25
		12,1	17,7	18,5	16,7	12,8	8,2	11,7	11,6	10,4	10,5	12,9	12,3	5,26
Standard deviation dB	Follicular ovulatory luteous	6,67	6,45	5,52	6,77	5,79	6	7,34	8,43	7,79	7,09	5,99	7,44	10,4
		6,93	6,59	5,31	6,65	5,94	5,9	7,09	7,19	7,09	6,74	5,91	8,14	10,9
		6,31	6,73	6,27	6,57	5,86	6,5	7,34	9,34	7,09	6,12	8,28	7,32	9,65

## DISCUSSION

Transient OAE may be detected in subjects with auditory thresholds up to 30 dB, and distortion product OAE may be detected in subjects with auditory thresholds up to 45/50 dB.^14^ DPOAE amplitudes were within the means presented in recent papers except at 6 kHz, where values were significantly below (over 9 dB) the mean.^14^^,^^15^ TOAE amplitudes were slightly above the means published in the literature, as seen when comparing the right and left ear means in the present study with the general TOAE mean in young adults published in 2000.^14^

Short-term hormone variations cause changes across a woman's body. These changes may be responsible for inner ear conditions such as Ménière's disease.^8^ In the current study, however, these variations did not result in significant changes in OAE responses; this result has already been reported in the literature.^12^

Differently from Coube and Costa Filho's^15^ assessment of distortion product OAE in 100 subjects showing no difference between ears, we found a significant difference in distortion product OAE at 1.5 kHz between right and left ears, where the former had higher values. This difference may not be particularly relevant for this test, as this frequency is usually little assessed due to noise interference from breathing, swallowing, vascular pulse and muscle movements.^15^ Another published paper16 has shown a noise peak between 1 and 1.5 kHz (mean 2.3 dB NPS) in distortion product OAE in audiologically normal neonates.

There were lower transient OAE amplitudes at 1 and 4 Hz across the menstrual cycle; lower distortion product OAE values were found at 1 and 6 kHz (the first and last tested frequencies). This finding may be explained at low frequencies, where it may be difficult to separate emissions from background noise, probably due to the cochlear tonotopic distribution.^14^ On the other hand, higher-voltage speakers are needed for identifying responses at high frequencies, making these responses harder to detect.^17^

Our sample included women aged between 20 and 35 years, having a regular hormonal cycle and normal hearing. It is possible for hormonal cycle phases not to have presented significant differences, as both transient and distortion product OAE may be detected in normal-hearing subjects. The absence of response amplitude changes during the menstrual cycle may seem surprising, as previous studies have detected auditory function differences in other forms of testing, such as dichotic listening tests5,6 or spontaneous OAE testing.^13^ Changes in auditory responses, however, appear to reflect central nervous activity, and OAE originate from pre-neural activity.^12^ Female-specific physiological events cause no significant differences in OAE responses, meaning that it is unnecessary to take the phase of the menstrual cycle into account when interpreting OAE test results.^12^ Response reliability is stable, regardless of the patient's hormonal cycle phase.

Subjects in our sample did not use anovulatory contraceptives during the study; prolonged use of these drugs may cause high-frequency sensorineural auditory loss.^18^ Other hormone changes, such as amenorrhea and menopause also affect the dynamics of spontaneous OAE frequency fluctuations.^13^

Our sample was small, which may have contributed to the non-significance of the results. Each woman was asked to visit the School Clinic three times, once during each menstrual cycle phase. Many evaluations were lost, as some of the subjects did not return for the last test; it was, therefore, hard to conclude the study within the available time. The number of participants in similar studies has also been small,^6^^,^^12^ underlining the methodological and logistical difficulty of this type of investigation.

## CONCLUSION

Based on this study we concluded that there were no significant differences in transient and distortion product OAE according to hormonal cycle phases.

## FINAL COMMENTS

We suggest that a similar study be undertaken including a larger sample and different inclusion criteria, such as women taking anovulatory contraceptives and women of different ages, to assess the effect of the hormonal cycle in theses situations.
